# A methodological framework for the efficient characterization of peripheral nerve stimulation parameters

**DOI:** 10.1088/1741-2552/ae0d31

**Published:** 2025-10-27

**Authors:** Rachel S Jakes, Benjamin J Alexander, Vlad Marcu, A Bolu Ajiboye, Dustin J Tyler

**Affiliations:** 1 Department of Biomedical Engineering, Case Western Reserve University, Cleveland, OH 44106, United States of America; 2 Human Fusions Institue, Case Western Reserve University, Cleveland, OH 44106, United States of America; 3 Louis Stokes Cleveland Veterans Affairs Medical Center, Cleveland, OH 44106, United States of America

**Keywords:** functional electrical stimulation, sensory restoration, peripheral nerve stimulation, neuroprostheses, strength-duration curve, human subjects research

## Abstract

*Objective.* Restoring movement and somatosensation with peripheral nerve stimulation (PNS) requires precise neural activation. Because pulse amplitude (PA) and pulse width (PW) recruit axons differently, intentionally modulating both could enable more advanced PNS. However, mapping the PA-PW space is currently prohibitively time-intensive. This paper proposes and clinically validates an efficient method to characterize multiple intensities in the PA-PW space for motor and perceptual sensory applications using minimal data collection. *Approach.* We used cuff electrodes implanted in one participant with a spinal cord injury to generate iso-EMG activation contours and two participants with upper limb loss to generate somatosensory perceptual iso-intensity contours in the PA-PW space. Strength-duration (SD) curves were mapped to the contours using varying sample point subsets and assessed for fit quality. Finite element modeling of a human nerve and activation simulations evaluated differences in recruited axon populations across the PA-PW space. *Main results.* SD curves accurately fit all levels of motor activation and perceptual intensity (median ${R^2}\, = \,0.996$ and $0.984$, respectively). Reliable estimates of SD curves at any intensity require only two sufficiently-spaced points (motor *$R^2\;=\;0.991$*, sensory *$R^2\;=\;0.977$*). Using this data, we present and validate a novel method for efficiently characterizing the PA-PW space using SD curves, including a metric that quantifies mapping accuracy based on two sampled points. *In silico*, intensity-matched high-PW and high-PA stimulation recruited overlapping, but not equivalent, axon sets, with high-PA stimuli preferentially recruiting large-diameter fibers and axons farther from the contact. *Significance.* This method enables rapid, accurate mapping of the stimulation parameter space for clinical motor and sensory PNS. The efficiency of the proposed characterization approach enhances the clinical feasibility of multiparameter modulation, establishing a framework for further exploration of two-parameter modulation for increased selectivity and resolution, reduced fatigue, and unique percept generation. (ClinicalTrials.gov ID NCT03898804).

## 1. Introduction

Peripheral nerve stimulation (PNS), or the use of electricity to activate peripheral nerves, is a powerful tool with the capacity for regulation and restoration of neurological function. For patients with spinal cord injuries or stroke, a type of PNS called functional electrical stimulation (FES) can circumvent the injuries in the central nervous system by functionally activating the peripheral motor neurons that innervate relevant muscles [1, 2]. For people with limb loss, sensory PNS can activate the neurons that formerly innervated the missing limb, reestablishing the sense of touch for sensory neuroprostheses [3]. For these and other PNS applications, modulation of the magnitude of the nervous system’s output is vital. Dexterous motor control requires the ability to smoothly adjust the activation of multiple muscles, while the ability to distinguish between light touch and a firm grasp is critical for handling fragile objects or holding someone’s hand. The magnitude of the neural response at the level of muscle activation and somatosensory perception is a function of both how many axons are activated and how often those axons fire. The number of axons that fire is modulated by both the pulse amplitude (PA) and the pulse width (PW) of the electrical stimulus.

PA and PW act as the *x*- and *y*-axes of a three-dimensional (3D) space, with the degree of axonal activation as the *z*-axis. Historically, charge-modulated intensity has been mapped and modulated in one dimension, varying PA or PW and holding the other parameter constant. While this method does modulate intensity, its one-dimensional axis limits the slope and maximum of certain axon populations’ activation. Often, multiple parameter combinations must be tested to find settings with appropriate characteristics. Full characterization of both parameters expands the control over which axonal populations can be activated and increases ability to recruit all possible sets of axons. Judicious selection based on full PA-PW space could produce shallower paths from threshold to maximum, which has long been sought after to enable precise movements [4–7] or improved compliance and shape detection [8]. Prior *in silico* [9, 10] and *in vivo* [10, 11] work has also shown that high-PA, low-PW stimulation can activate different axonal populations than low-PA, high-PW stimulation. A clinical study also showed differential activation of the Hoffman reflex (H-reflex) at matching M-wave magnitudes across different PA and PW paradigms [12]. If these differences in axonal activation are substantial enough, characterization of the full parameter space becomes even more clinically useful. For motor control, increased inter-muscle selectivity would result in improved dexterity, while improved intra-muscle selectivity, or the ability to activate different motor units within the same muscle, would be highly beneficial in minimizing fatigue [13–16]. For sensory restoration, more specific control of axon population recruitment could enable the modulation of percept location with a single contact, independent of intensity. For both motor FES and sensory PNS, multidimensional charge modulation may increase the usable range of intensity by identifying how pain or contraction thresholds change across the space. While the functional potential of a two-dimensional charge modulation space is promising, the primary barrier to utilizing the full extent of this charge modulation space is the time to calibrate stimulation in both dimensions.

Because the field lacks an efficient framework for PA-PW recruitment, charge is typically modulated via either PA or PW control while holding the other parameter constant. For motor neuroprostheses, recruitment curves have been the field standard for decades [17–23]. However, each recruitment curve provides only a single slice of the complete stimulation space. Since they have been shown to have inconsistent shapes as the sum of multiple sigmoidal curves [21], 5–20 points are usually needed to characterize a single curve. Furthermore, multiple recruitment curves from a given contact are often necessary to optimize elements such as the slope, range, and selectivity of muscle activation. For sensory neuroprostheses, while perceptual threshold is sometimes characterized in two dimensions [24–26], standard intensity modulation procedures typically choose either PW or PA modulation and keep the other constant, as defined by the charge term of the activation charge rate equation [27–30]. In cases where multiple parameters are explored, calibration is completed by repeatedly sampling one parameter at preset values of another, e.g. increasing PA until perception is achieved at five preset PWs [25, 26]. This method is both time-consuming in comparison to single-parameter calibration and misses information about the spaces between samples. Further, estimation of equal perceptual intensities in multiple dimensions above perceptual threshold with established calibration methods is limited, with studies either only investigating both PA and PW at threshold [24, 25] or ultimately choosing only one parameter for modulation above threshold [25, 26]. Pulse frequency (PF) is also sometimes used to modulate sensory intensity, but PF impacts perceived intensity less than charge [27, 29] and additionally alters sensation quality [28, 31]. Therefore, frequency modulation is less suitable than charge modulation for independent intensity control.

In both sensory and motor neuroprostheses, a key reason for the lack of simultaneous PA-PW modulation is the prohibitive amount of time it would take to characterize the outputs of all possible parameter combinations. For motor FES, single-pulse twitch-activation can be used to approximate the motor output of continuous tetanic stimulation [32]. By measuring force [33] or electromyographic [20] twitch outputs it is then possible to quickly characterize muscle responses to electrical stimulation. However, for systems with many multi-contact electrodes, even this methodology can quickly become prohibitively time-consuming. A method for efficient characterization based on a 3D extrapolation of the Gompertz equation has been described [34], but it has not been benchmarked against any other methods and has not been widely used. To our knowledge, no other methods have been established in the literature to characterize the full PA-PW-motor stimulation space using naïve sampling, polynomial fitting, machine learning, or other strategies.

For sensory activation, the duration of stimulation-space mapping is an even greater challenge due to the required human-in-the-loop aspect of sensory percept characterization. As an added complication, single pulse and continuous stimulation trains have been shown to evoke different percepts, so stimulation trains must be used for functional percepts for sensory neuroprostheses [25]. For motor and sensory neuroprostheses to make use of the full PA-PW stimulation space, a faster method of characterization is necessary.

Our proposed method for efficient multi-dimensional stimulation space characterization is to decrease the necessary number of sampled points by applying the well-validated neural feature, the strength-duration (SD) curve (equation (1)),

\begin{equation*}{\text{PA}} = {\text{P}}{{\text{A}}_{{\text{rh}}}}*\left( {1 + \frac{{{\text{P}}{{\text{W}}_{{\text{ch}}}}}}{{{\text{PW}}}}} \right)\end{equation*}

where PA_rh_ is the rheobase and PW_ch_ is the chronaxie. The equation describes the relationship between the PA (strength) and PW (duration) of a square wave required to create the same level of evoked neural activation. The rheobase is the amplitude required for activation at infinite PW, while the chronaxie is the PW on the curve when the PA is double the rheobase. The SD curve was first described as an axon-level characteristic of the intracellular threshold of activation by Weiss in 1901 [35]. Though other models have been proposed, the Weiss model (equation (1)) has been shown to be the most accurate *in silico* and *in vivo* [36–38]. It has since been demonstrated that Weiss’ SD curve accurately scales from the single fiber response that it was initially defined for to a collective response at multiple levels of activation as quantified by compound muscle and compound sensory action potentials (CSAPs) [37, 39]. In addition to the objective measurement of CSAPs, sensory perception induced by PNS has been shown to obey this same relationship at the threshold of activation [24, 40, 41]. However, it remains unknown if SD curves can accurately describe perceptual intensities at higher percept magnitudes across the dynamic intensity range.

Given the apparent robustness of the SD relationship and the fact that the equation has only two parameters, it has been suggested that ten, five, or even two points may be sufficient to accurately compute the entire curve [24, 37]. Recent *in silico* work, based on the Weiss equation, suggested that the furthest possible two points on the curve could be the optimal samples to accurately construct the curve [42]. No modeling work involving axon models or neural channel dynamics have validated these results. Further, no *in vivo* research has investigated the ideal points to fit motor and somatosensory SD curves or how the accuracy of these fits varies with different parameter pairs or levels of activation.

Establishing the SD curve as a reliable tool for PNS parameter space characterization requires examining several key physiological and methodological factors. Firstly, the SD curve must be a consistently accurate representation of the system neural activation that we are interested in characterizing. We therefore demonstrate that the SD curve equation accurately fits evoked EMG and perceived intensities measured from human participants across the full functional range of activation. Secondly, optimizing SD curve sampling efficiency and accuracy is essential for its application in FES and sensory PNS activation. To address this, we identified the most effective points for constructing SD curves efficiently and assessed the robustness of curve generation when using non-optimal samples. Finally, since a major potential benefit of the combined PA and PW modulation is the ability to activate different axonal populations, we used *in silico* models of the human nerve to characterize the differences in the populations activated at high-PA and high-PW conditions while maintaining a similar number of activated fibers.

## 2. Methods

### 2.1. Clinical

#### 2.1.1. Motor

##### 2.1.1.1. Participant

One male in his thirties with a motor complete C4 level spinal cord injury participated in motor data collection as part of the ‘Reconnecting the Hand and Arm to the Brain’ clinical trial [43]. Eight composite flat interface nerve electrodes (C-FINEs) [44, 45] were implanted around the participant’s median, radial, ulnar, musculocutaneous, lateral pectoral, and axillary, suprascapular, and long thoracic nerves 5 years post injury. Data collection for this paper started four years after implantation and consisted of six four-hour sessions that occurred over four months. The US Food and Drug Administration approved an Investigational Device Exemption (ClinicalTrials.gov ID: NCT03898804) for the use of the peripheral nerve electrodes in this study. The University Hospitals Cleveland Medical Center institutional review board approved the protocol and provided study oversight. The participant provided informed consent for the study.

##### 2.1.1.2. Stimulation delivery and calibration

A Windows PC running a custom binary search method on MATLAB 2024a (*MathWorks, Natick, MA, US)* determined stimulation parameters and sent them to the stimulator through an intermediary xPC realtime target. Electrical stimulation was provided by a custom current-controlled universal external control unit (UECU) stimulator [46]. The PW resolution of the stimulator was 1 *µ*s from 1 to 250 *µ*s. The PA resolution of the stimulator was 0.1 mA below 2 mA and 1 mA at higher amplitudes. The stimulator delivered single pulses of a cathode-first charge-balanced biphasic square wave between one of the cathodic contacts and the anode strips on the C-FINE. We quantified muscle activation using 3 trial-averaged EMG responses rectified and integrated from 5 to 45 ms after the stimulation pulse [20]. The Bluetooth-enabled surface EMG sensors of the Trigno Wireless Biofeedback System (*Delsys, Natick, MA, USA*) recorded EMG and provided realtime feedback to the active search method.

**Figure 1.**
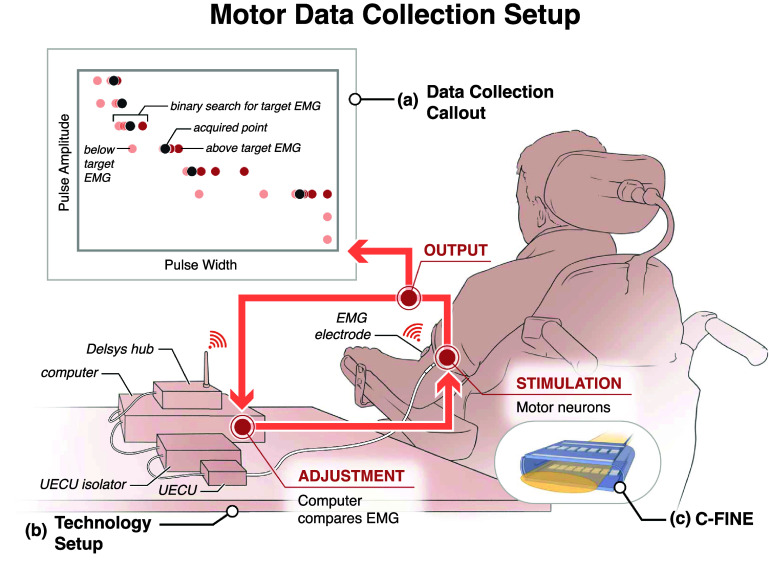
Experimental setup for muscle activation contour collection. (a) Equal intensity muscle activation contours were acquired using a binary search algorithm that determined the optimal stimulation parameters to match a target EMG level. The binary search method started at a pulse amplitude (PA) of 0.1 mA and pulse width (PW) of 250 *µ*s. The computer increased the PA until the recorded EMG value was above the target EMG level. At this point, the computer performed binary search to find the PW that provided the closest EMG value to the target. A repeated binary search at increasing amplitudes through all possible PAs completed the contour. (b) The UECU stimulator provided electrical stimulation to the nerve cuff electrodes in the participant’s arm. A Delsys Trigno EMG sensor recorded the EMG voltage which the computer then processed. (c) Stimulation was delivered through C-FINE cuffs containing 15 cathodic contacts and an anode strip.

On each of five different nerve cuff electrodes (figure 1(c)), we tested two separate cathodic contacts, using the built in anode strip as the anode and recording EMG from one large and functionally relevant muscle or muscle group innervated by the given nerve (table 1).

**Table 1.** Motor nerve cuffs and muscles analyzed. Five nerve cuffs, each on a different nerve in the arm or shoulder, were tested for the acquisition of muscle activation data. For each nerve cuff, one large muscle or muscle group that the nerve is known to innervate was recorded from using surface EMG. Two different electrode contacts were tested for each nerve cuff, one on the top of the cuff and one on the bottom.

**Nerve cuff**	Axillary	Lateral pectoral	Musculocutaneous	Median	Radial
**Muscle(s) recorded from**	Posterior deltoid	Upper pectoralis	Biceps	Wrist and finger extrinsic flexors	Wrist and finger extrinsic extensors

##### 2.1.1.3. Equal muscle activation contour generation

Our search algorithm obtained equal muscle activation contours that mapped the relationship between PA and PW and their relative effect on the resulting muscle activation. For each contour, a specific target EMG value was defined and our experimental setup (figure 1) used binary search to acquire the set of PA-PW pairs that resulted in the EMG values that were closest to that target. We acquired these contours for 10%, 30%, 50%, 70%, and 90% of the maximum evoked muscle activation for 10 different muscle-contact pairs. These maxima were established prior to data collection by finding the upper asymptote of a PW-modulated recruitment curve for each muscle-contact pair.

Our binary search method (figure 1) acquired each contour by starting at 0.1 mA and 250 *µ*s and increasing PA in steps of 0.1 mA until it recorded an EMG value above the target threshold. At this point the method varied PW from 1 to 250 *µ*s using binary search until it determined the PW value that resulted in muscle activation closest to the target level. The sampling method then moved up to the next PA value, using the final PW value from the previous PA as the new maximum stimulus value. We updated the maximum allowable PW in this way to prevent painful, high intensity stimuli and with the assumption that increased PA and identical PW should always produce the same or greater levels of muscle activation. The system continued this process until the maximum PA value of 3.0 mA was reached. We acquired a total of three blocks, each consisting of all five activation levels with the order of the activation levels randomized within each block to remove any dependence on order.

#### 2.1.2. Sensory

##### 2.1.2.1. Participants

Two participants (both male) with upper limb loss participated in sensory data collection. Subject 1 underwent a left transradial amputation from traumatic injury 11 years prior to this study and was implanted with two 16-channel C-FINEs around the median and ulnar nerves in the residuum 3 years later, 8 years prior to data collection for this study. Subject 2 underwent a right transhumeral amputation from a traumatic injury also 11 years prior to this study and was implanted with two 16-channel C-FINEs around the median and radial nerves in the ipsilateral axilla 7 months prior to data collection for this study. The sensory data in this paper comprises one four-hour session with Subject 1 and three four-hour sessions with Subject 2 over four months, all sampled from the median cuff. Median nerve stimulation could elicit both motor and sensory percepts in Subject 1 but only sensory percepts in Subject 2 due to the absence of the corresponding innervated muscles. The US Food and Drug Administration Investigational Device Exemption, the Cleveland Department of Veterans Affairs Medical Center Institutional Review Board, and the Department of the Navy Human Research Protection Program approved all study devices and procedures. All participants provided informed consent for the study.

##### 2.1.2.2. Stimulation delivery and calibration

All stimuli were 100 Hz trains of biphasic cathode-first charge-balanced square wave pulses between one of 15 cathodic contacts and an anode strip on the median nerve C-FINE. Stimulation parameters set in MATLAB R2020a (*MathWorks, Natick, MA, USA*) drove a current-controlled Grapevine Neural Interface Processor (*Ripple Neuro, Salt Lake City, UT, USA*).

To assess each participant’s approximate perceptual range at a long PW (250 *µ*s), we applied an increasing PA staircase method with two reversals and a step size of 0.01 mA. Participants assigned the perceptual threshold and upper bound intensity numbers via the magnitude estimation methodology described by Graczyk *et al* [27]. The upper bound was defined as the first of either maximum comfortable sensation or a muscle contraction. Participants also identified perceptual threshold and maximum comfortable intensity via an increasing PW staircase method at a PA of 2.0 mA. Last, participants labeled PAs at a PW of 250 *µ*s that produced sensations at about 25%, 50%, and 75% of their dynamic intensity range as defined by their minimum and maximum magnitude estimations. These five PAs across the dynamic intensity range became the reference values for equal intensity contour generation. Over the course of the session, the estimated magnitude of these reference stimuli sometimes shifted, but the stimulation parameters remained constant as long as the participant still reported the stimuli as perceptible and comfortable.

##### 2.1.2.3. Equal somatosensory perceptual intensity contour generation

**Figure 2.**
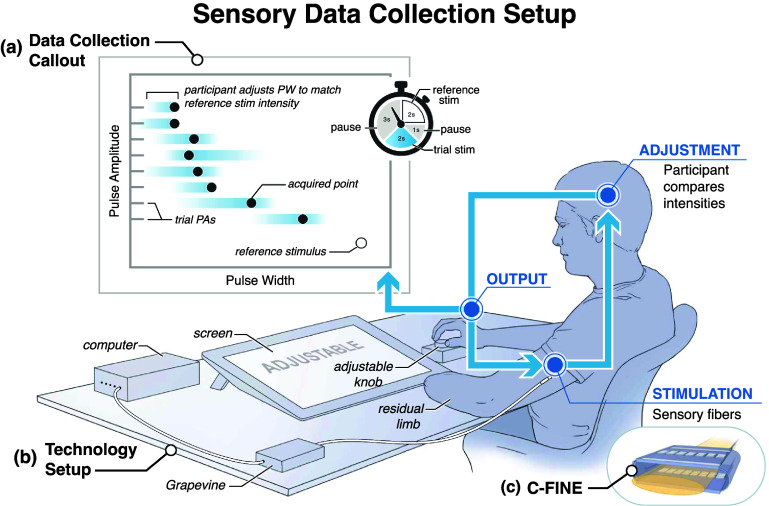
Experimental setup for sensory perceptual intensity contour collection. (a) Equal intensity contours were collected using a method of adjustment, in which participants used an adjustable knob attached to a rotary encoder to adjust pulse width at set pulse amplitudes to match the intensity of a reference stimulus. Reference and trial stimuli were alternated as shown in the timer icon. (b) The Ripple Grapevine provided stimulation through percutaneous wires in the participant’s residual limb to a cuff electrode around the participant’s median nerve. (c) Stimulation was delivered through C-FINE cuffs containing 15 cathodic contacts and an anode strip.

Equal intensity contours map the relationship between two variables and their relative effect on perceived intensity. In haptic feedback research, equal intensity contours have been generated between frequency and amplitude of a signal using the method of adjustment [47, 48]. Our experimental design (figure 2) modifies those methods by determining the PWs at which two different PAs of stimulation feel equal in intensity.

We defined a single intensity contour as nine points: the reference PA and eight trial PAs sampled above the highest reference PA for the contact. One intensity contour was created per reference stimulus per block. To generate an equal intensity point, participants received two seconds of a reference stimulus, i.e. a reference PA at 250 *µ*s PW, followed by two seconds of a trial stimulus, defined as a trial PA and an adjustable PW. A 1 s and 3 s pause followed the reference and trial stimuli, respectively, to minimize adaptation, as recommended by Van Doren [47]. Participants used a rotary encoder knob to adjust the PW of the trial stimulus. The participant could turn the PW knob at any point during the trial and the trial PW would update to the new value the next time the trial stimulus was provided. This pair of stimuli repeated until the participant felt the reference and trial stimuli were identical in intensity. To account for anchoring bias, the adjustable PW started at or below perceptual threshold for each trial. We also placed limitations on the adjustable stimulation that ensured participant safety while still providing sufficient modulation space for participants to place the trial stimulus anywhere in their dynamic range.

Each equal intensity contour was collected in a randomized order over three one-hour blocks for a total of 27 points per contour. In some cases, full contours could not be collected for weaker intensities due to adaptation in the later blocks. Contours reported in this manuscript had a minimum of three trials per reference and trial stimulus pair and a minimum of nine points per contour per block. In all, three full contours were collected from contact 2 on Subject 1, four contours were collected from contacts 1 and 6 on Subject 2, and five contours were collected from contact 5 on Subject 2. Some contours from contact 5 on Subject 2 had four or five trials per reference and trial stimulus pair, as repeat points were collected in the final block to better investigate any temporal effects.

#### 2.1.3. Data analysis

For both motor and sensory equal intensity contours, we first assessed if there was any significant difference across points accumulated in different blocks for the same equal intensity contour to determine if points should be grouped or separated by block for curve fitting. Two non-linear mixed effects models fit with the Weiss equation (equation (1)) were applied to each equal intensity contour, one accounting for block number as a random effect and one without random effects. The fits were compared with a likelihood ratio test. If block number had a significant effect on the model, curve fits would be completed individually for each block. Otherwise, blocks would be grouped together for curve fits. In accordance with this methodology, motor contours from different blocks were treated as unique contours, while sensory contours were combined across blocks and treated as samples of the same contour.

**Figure 3.**
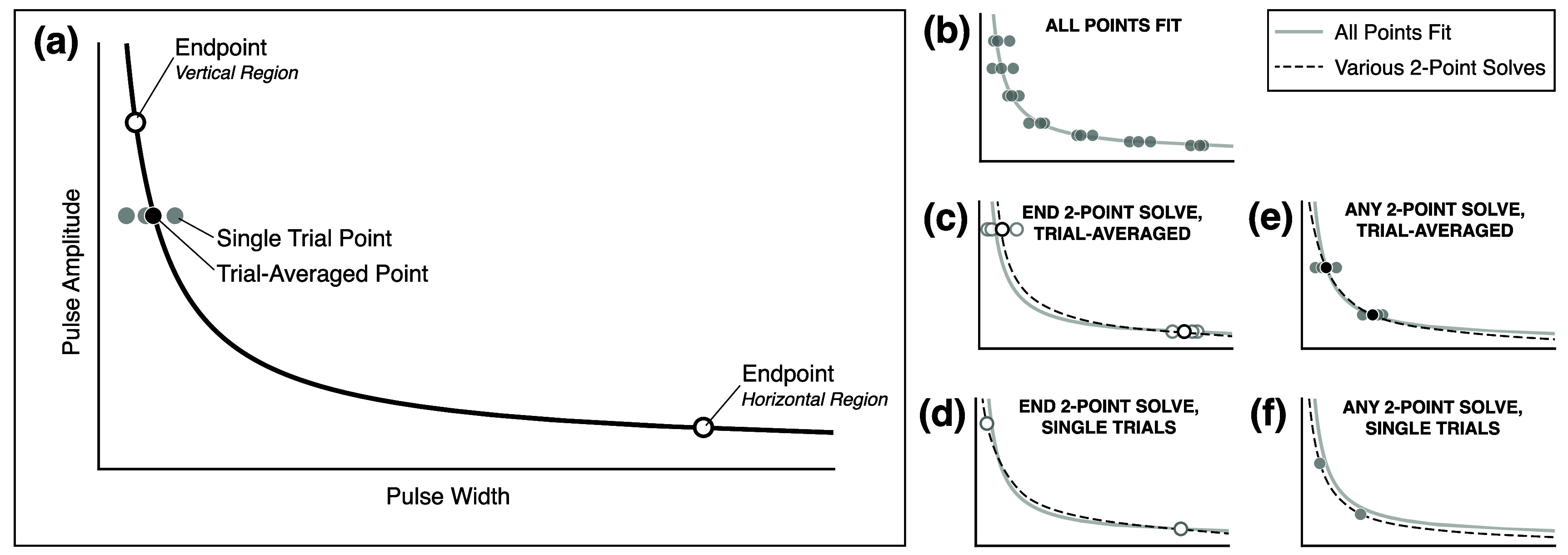
Graphical representation of common terms and fit strategies. Throughout the figure, solid lines represent a curve fit with all sampled points, while dashed lines show illustrative SD curves solved with two points. Endpoints are shown in open circles while non-endpoints are solid. Trial-averaged points are shown in black, while points from a single trial are in gray. (a) Endpoints are the two points sampled at the most extreme ends in the PA-PW space, one where the slope is most horizontal and the other the most vertical. Trial-averaged points are the mean PW of three trials at the same PA and intensity. (b) All points from each equal intensity contour were fit to an SD curve to assess how well SD curves describe the contours. (c) Trial-averaged endpoints were used to solve equation (1). This fit strategy was only used in sensory data analysis, as the motor data was not trial-averaged. (d) Equation (1) was solved with single trials of endpoints, one each from the vertical and horizontal regions. All possible combinations of single trials were used, e.g. if there were three trials at each endpoint, nine pairs of points and solutions were assessed. (e) Trial-averages of the points from any two sampled PAs in the contour were applied to equation (1). Like the trial-averaged endpoint fit strategy, this technique was only assessed with sensory data. (f) Any two points with different PAs were used to fit equation (1). All possible pairs of points with distinct PAs were assessed.

To assess the ability of the Weiss SD curve to describe the PA-PW equal intensity contours, we applied equation (1) to each contour using a non-linear model with an iterative least squares estimation fit algorithm (figure 3(b)). Comparing the resulting curve to the points in the equal intensity contour yielded a goodness-of-fit *R*^2^ value.

To evaluate if the SD curve solved as a system of equations with just two (PW, PA) points could accurately describe an equal intensity contour, an endpoint method and any two-point fit method was used (figures 3(a) and (c)–(f)). Previous mathematical modeling suggests that the points sampled furthest apart in the PA-PW space, one each in the vertical and horizontal regions of the curve (i.e. the ‘endpoints’), have the highest probability of returning a good fit (37). The any-two-point method was used as a comparison because the position of the ‘endpoints’ is somewhat arbitrary. In the motor analysis, no trial averaging was done across contours, while in the sensory analysis, both methods were tested with and without trial averaging of the solver points (figures 3(c)–(f)). In all cases the resulting models were compared to the sampled equal intensity contour via an *R*^2^ goodness-of-fit calculation.

A Kolmogorov–Smirnov test assessed the normality of each *R*^2^ distribution. As the distributions were non-normal, a Kruskal–Wallis test compared *R*^2^ values across three motor and five sensory distributions to assess differences in goodness of fit based on the number and selection of points used to create the SD curve. Non-parametric Dunn-Šidák tests with multiple comparison corrections followed.

**Figure 4.**
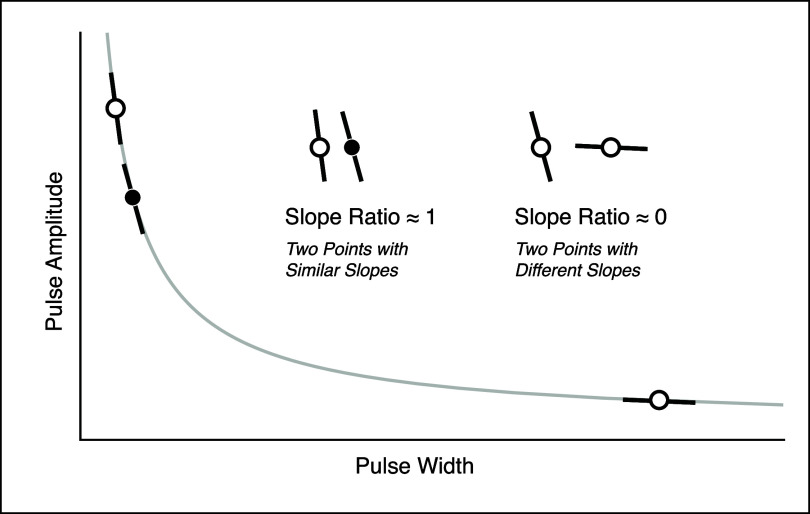
Slope ratio analysis visualization. Three sample points, two of which are endpoints, are shown with their respective tangent lines. The ratio of the tangent line slopes of the points in the same region of the curve is near to one, while the slope ratio of the tangent lines of the two endpoints in different regions approaches 0. The slope ratio is always calculated with the lower PA point, or the point closer to the horizontal region of the curve (figure 3(a)), in the numerator.

Last, we identified the threshold at which fit accuracy is lost based on selection of solver points. First, we used the derivative of the full fit SD curve at the PA of each solver point (equation (2)) to normalize the relative location of each point. We then plotted goodness of fits as a function of the ratio of the slopes, or slope ratio (SR), of the SD curve at the two solver points for both the motor and sensory data sets (equation (3); figure 4),

\begin{equation*}\frac{{{\text{dPA}}}}{{{\text{dPW}}}} = - \frac{{{\text{P}}{{\text{A}}_{{\text{rh}}}}\,*\,{\text{P}}{{\text{W}}_{{\text{ch}}}}}}{{{\text{P}}{{\text{W}}^{\text{2}}}}}\end{equation*}

\begin{equation*}{\text{SR}} = \frac{{\frac{{{\text{dPA}}}}{{{\text{dPW}}}}{|_{{\text{PA = P}}{{\text{A}}_i}}}}}{{\frac{{{\text{dPA}}}}{{{\text{dPW}}}}{|_{{\text{PA = P}}{{\text{A}}_j}}}}},{\text{ P}}{{\text{A}}_i} &lt; {\text{ P}}{{\text{A}}_j}.\end{equation*}

Because of the positive concavity of the SD curve, SR will always be between 0 and 1, with points in different regions of the curve yielding an SR closer to 0 and points in the same region approaching an SR of 1. SR is also unitless so it can be standardized across paradigms and sample spaces.

### 2.2. Modeling

We used simulations of PNS to examine differences in axonal activation at high-PA and high-PW stimulation at equal intensities of activation. Finite element models based on the histology of human radial nerve samples provided estimations of axon population activation. Electrode configurations based on 16-contact C-FINEs [44, 45] were placed in the model with 0.4 mm diameter circular contacts and an overall model dimension of 10 by 1.5 mm. The conductivities of the different neural tissues were assigned in accordance with empirical data on the resistivities of the endoneurium [49], perineurium [50], and epineurium [51], as well as saline [52] (table 2). To determine voltage along different axons, a 1 mA current was simulated at each of the 16 contacts, and the resulting voltage fields were recorded within each fascicle. All simulated stimuli were biphasic square currents with a 2:1 charge recovery ratio and no inter-pulse interval.

**Table 2.** Conductivities of simulated tissues and materials in FEM.

Material	Conductivity (S m^−1^)	
Endoneurium	8.23 * 10^−2^ (transverse)	[49]
	5.71 * 10^−1^ (longitudinal)	
Perineurium	2.1 * 10^−3^	[50]
Epineurium	8.26 * 10^−2^	[51]
Saline	2	[52]
Silicone	1 * 10^−7^	[52]

Simulating the axonal population response to a given monopolar stimulus was done in MATLAB 2024a (*MathWorks, Natick, MA, USA*) by solving the Gaines cable model [53] in response to externally applied voltage fields as solved for in COMSOL (*COMSOL, Burlington, MA, USA*). The Gaines model captures differences in ion channel densities between motor and sensory axons, enabling the identification of distinct axonal dynamics. Each simulated fascicle contained 700 axons with randomized internode offsets, positions, and physiologically informed diameters based on sensory and motor populations. To vary the stimulation PA, we linearly scaled the COMSOL voltage fields from the 1 mA template. This PA modulation method aligns with previous studies that suggest peripheral neural tissue is electrically linear with minimal voltage-dependent impedance changes [54–56]. We simulated axonal responses to different PWs by varying the duration of external voltage application in the Gaines model.

The simulation model distinguishes sensory and motor axons by assigning each axon population distinct channel conductances [53] and axon diameter distributions. We assign sensory axon diameters based on the distribution of the human sural nerve [57], as it is a well-characterized sensory peripheral nerve and has served as a fiber distribution template in other PNS models [58]. The motor axon diameter distribution was obtained by starting with the human median nerve axon distribution [59] and proportionally subtracting the sural nerve distribution, thus isolating only the motor axon diameter distribution. Multiple SD curves were generated by simulating monopolar stimulation along all 15 stimulating contacts of the simulated C-FINE at nine PAs between 0.1 and 0.6 mA and ten PWs between 10 and 250 *µ*s. We generated an exhaustive set of PA and PW pairs and recorded their corresponding neural activations. We then grouped stimuli into ‘iso-intensities,’ with each stimulus in an iso-intensity activating within 1% of the same total axon count. We classified stimuli as in the vertical region of the SD curve if their PWs were 50 *µ*s or less and as in the horizontal region of the curve if they were 100 *µ*s or more. We compared the resulting iso-intensities to the SD curve (equation (1)) by calculating *R*^2^ values of the Weiss equation fits to iso-intensities in both the motor and sensory nerve.

Using the simulated data, we measured the axon diameters uniquely activated between stimulation in the vertical and horizontal regions of the SD curve. We also quantified the distances from activated axons to the stimulating contacts under these conditions. T-tests were used to determine if axon diameters and average axon-to-contact distances differ significantly between the horizontal and vertical regions of the same SD curve. Finally, we compared the differences across SD curves that activate various percentages of the model’s axons and assess whether low-intensity stimulation exhibits trends distinct from high-intensity stimulation.

## 3. Results

### 3.1. Clinical

#### 3.1.1. SD curves describe equal EMG and perceptual intensity contours at high goodness of fit

**Figure 5.**
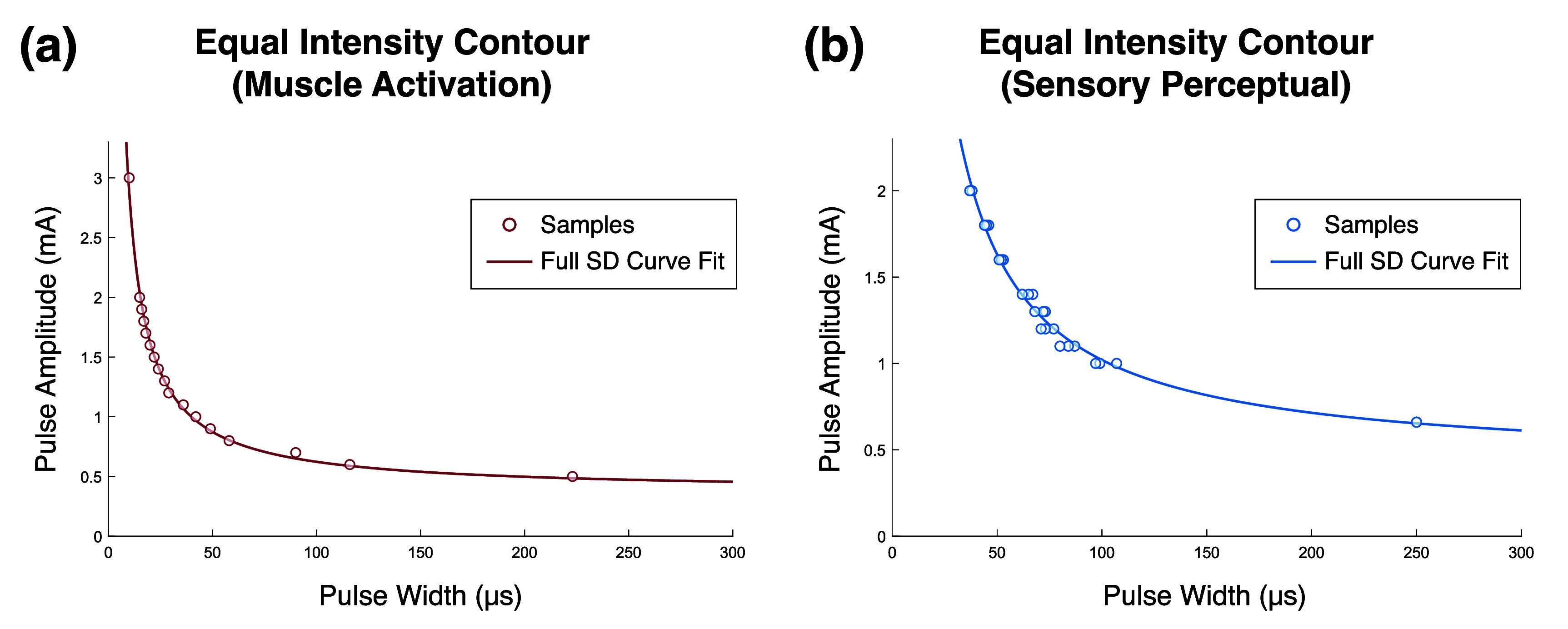
Equal intensity contours with SD curve fits. Data points collected for the equal intensity contours are shown in circles, while the Weiss SD curve (equation (1)) fit to those data is a solid line. (a) This contour was collected for 10% EMG activation of the biceps muscle of the motor participant. A total of 17 points at unique PAs were collected from a single block. The given SD curve fit has an *R*^2^ value of 0.995. (b) This contour was collected for a mid-intensity sensory percept from Subject 2, with a reference stimulus amplitude of 0.66 mA. Points at nine PAs were collected in three blocks for a total of 27 points. The given SD curve fit has an *R*^2^ value of 0.990.

The Weiss SD curve equation was found to describe equal muscle activation and perceptual intensity contours across the respective activation ranges, with median *R*^2^ values of 0.996 and 0.984, respectively (figure 5).

#### 3.1.2. Two points can accurately solve for SD curves at multiple levels of neural activation

**Figure 6.**
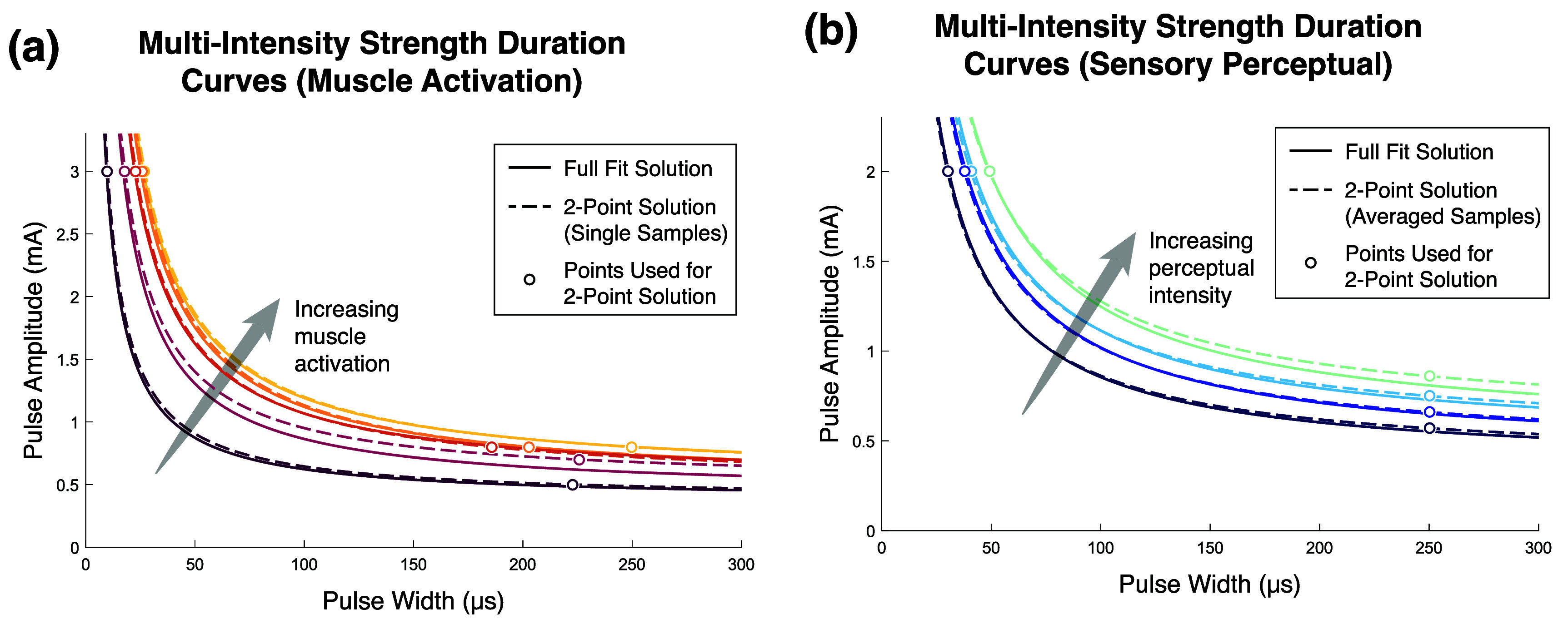
Multi-intensity fit and solved SD curves. SD curves that were fit with a full contour, as in figure 5, are shown with solid lines, while SD curves that were generated by solving for the Weiss equation with the two points that are furthest into the horizontal and vertical regions are shown with dashed lines. Lines of the same color correspond to the same contour. (a) These curves are generated from activation of the biceps muscle, with each contour representing 10%, 30%, 50%, 70%, or 90% of the asymptotic maximum of that muscle. (b) These curves are generated from activation of sensory fibers in the median nerve of Subject 2 that primarily result in percepts on the thumb and palm of the phantom limb. The curves shown span approximately 20% to 80% of the participant’s dynamic intensity range.

The two constants that define an SD curve, rheobase and chronaxie, can be found by solving the system of equations with two known points. These two-point solutions were solved using the points furthest into the horizontal and vertical regions of the SD curve and compared to the fit with the full data set (figure 6). In the case of the sensory data (figure 6(b)), because three samples were collected for the highest point in the vertical region, the average of those three values was used to solve the system of equations.

**Figure 7.**
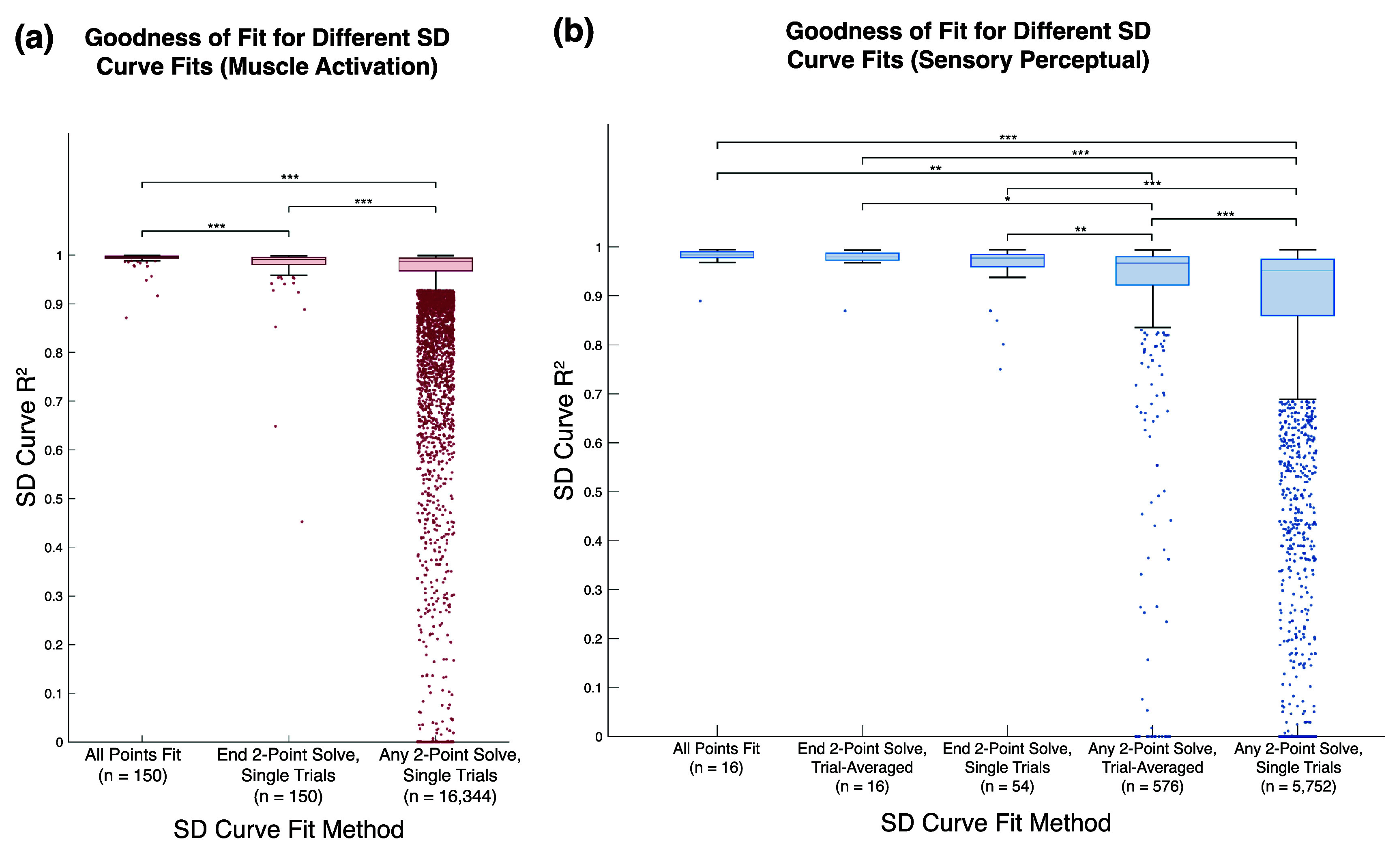
Distribution of *R*^2^ values by SD curve fit method. Outlying values, defined as values more than $1.5\ast IQR$ away from the 25th or 75th percentile, are jittered points below the boxes. Outliers below zero were set to 0 for visualization. Two-point pairs with no solution were set to an *R*^2^ of 0. Significant differences between groups as determined by a Dunn-Sidak post-hoc multiple comparison are shown with asterisks above pairings ($ ^{*} p\;\lt\;0.05$, $ ^{**} p\lt0.01$, $ ^{***} p\lt0.001$). (a) The muscle activation SD curves fit with full dataset, solved with endpoints, and solved with any two points have median *R*^2^ values of 0.996, 0.991, and 0.987 respectively. Outliers represent 10%, 9.3%, and 14.0% of each dataset respectively. (b) The sensory perceptual SD curves fit with the full multi-block dataset, solved with trial-averaged endpoints, solved with single-trial endpoints, solved with any two trial-averaged points, and solved with any two single-trial points have median *R*^2^ values of 0.984, 0.980, 0.977, 0.967, and 0.951, respectively. Outliers represent 6.3%, 6.3%, 7.4%, 14.6%, and 14.9% of each dataset, respectively.

To assess how robust the quality of the solved SD curve is to point selection and trial-by-trial variance, curves were fit with all contour points, the two endpoints, and every combination of two points in the contour. Due to small but significant differences between blocks for motor data, all motor SD curves were fit and evaluated with data from each block individually rather than combining across blocks (figure 7(a)). For sensory data, the two-point solved curves were calculated with both trial-averaged points and single-trial points and evaluated against data from all blocks for the given contour (figure 7(b)). Every fit method resulted in a median *R*^2^ > 0.95.

Because of the wide differences in group size and non-normal distribution of the *R*^2^ values, a Kruskal–Wallis test compared the goodness of fits of the groups. The Kruskal–Wallis test showed significant differences for both motor and sensory data sets (both $p &lt; 0.001$). Dunn-Šidák post-hoc tests showed significant differences between the distribution of *R*^2^ values for all SD curve solution methods for motor (all $p &lt; 0.001$). For sensory SD curves, significant differences were found between the fits using any two single-trial points and every other methodology ($p &lt; 0.001$). Differences with varying significance were also found between the solution with any two trial-averaged points and the other four methodologies. No significant difference was found between the goodness of fits of the SD curves fit with all points and the curves solved with trial-averaged or single-trial endpoints.

**Figure 8.**
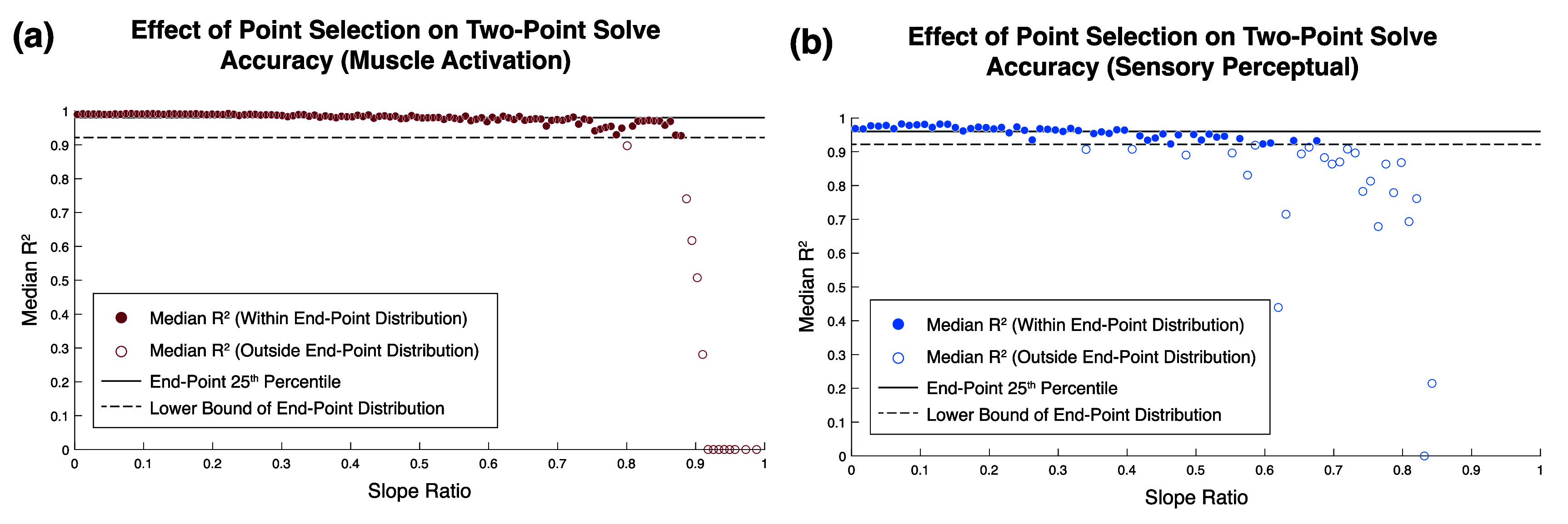
Two-point *R*^2^ fits by slope ratio at solver points. Point pairs with similar derivatives have a slope ratio closer to 1. Points were binned in the number of groups equal to the square root of the number of data points, with the width of each bin equal to the range of slope ratios divided by the number of bins. The *y*-coordinate is the median *R*^2^ value in the bin. The solid and dashed lines indicate the *R*^2^ values at the 25th percentile and whisker of the single-trial endpoint *R*^2^ distributions (figure 7). (a) The motor data was binned into 128 groups, with a bin width of 0.0078 in slope ratio. Endpoint 25th percentile and distribution boundary *R*^2^ were 0.980 and 0.958, respectively. (b) The sensory data was binned into 76 groups, with a bin width of 0.0111. The maximum slope ratio in the sensory data was 0.843 rather than 1 because the PA-PW space was sampled at a lower density. Endpoint 25th percentile and distribution boundary *R*^2^ were 0.960 and 0.922, respectively.

In comparing the *R*^2^ distributions of the single-trial solutions, the interquartile ranges of the any-point solutions are about two and four times wider than those of the endpoint solutions for muscle activation and sensory percepts, respectively. To further investigate the relationship between point selection and solution fit, *R*^2^ values are plotted as a function of the SR (equation (3); figure 8). Points sampled further apart on the curve, such as endpoints, have an SR closer to 0, while points sampled closer together have an SR closer to 1. To determine how far apart solver points must be on an SD curve to be classified as ‘endpoints’, the any-point data was compared to the endpoint *R*^2^ distribution. Solid and dashed horizontal lines indicate the 25th percentile and lower bound of the single-trial endpoint solution distributions, respectively. Points above these lines fall within the expected distribution of endpoint goodness of fit. Point pairs with ${\text{SR}} &lt; 0.68$ and ${\text{SR}} &lt; 0.34$ fall within the endpoint *R*^2^ distributions for muscle activation (figure 8(a)) and sensory perception (figure 8(b)), respectively. The highest SRs with a median goodness of fit within the endpoint *R*^2^ distribution were 0.86 and 0.68 for motor and somatosensation.

#### 3.1.3. Perceptual SD curves do not significantly change across time despite adaptation in magnitude estimation

**Figure 9.**
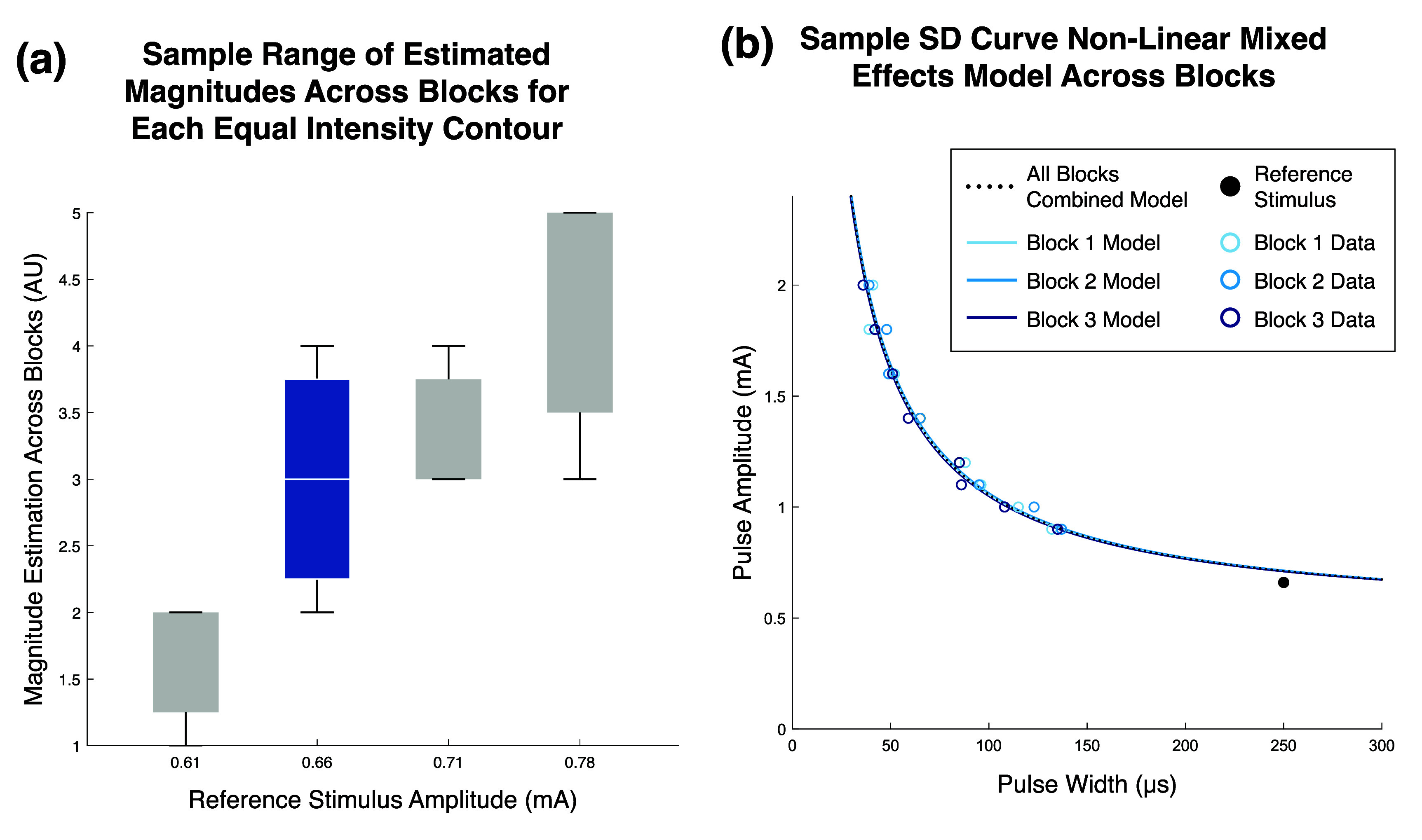
Changes in perceived sensation magnitude and SD curve fits across blocks. Data is from stimulation on contact 1 of Subject 2, which elicits sensation on the middle finger of the phantom limb. (a) Each box displays the range of magnitudes assigned to each equal intensity contour, denoted by the reference stimulus amplitude given on the *x*-axis, across three blocks. Each reference stimulus elicited a distinct perceptual magnitude during calibration but may have shifted to alternative magnitudes by the beginning of block 1 due to adaptation during the calibration phase. (b) The data and SD curves for the box in blue in subfigure (a) are shown here. Each solid-line single-block model was generated using a non-linear mixed effects model with the block number as the random effect. The dotted black line is the non-linear model calculated without accounting for differences in blocks. The curves generated from blocks 1, 2, and 3 were rated as having magnitudes of 4, 3, and 2, respectively. No significant difference was found between the within-block curves despite the difference in reported magnitudes.

For all 16 sensory iso-intensity contours, block number did not significantly affect the non-linear mixed effects model (figure 9(b)). This comparison includes the contours from the dataset collected across a mix of two standard 45 min blocks and one repetitive block in which each reference and trial stimulus pair point was collected three times back-to-back. Despite the absence of significant difference found in the SD curves for each intensity across blocks, the participants rated 14 out of the 16 contours as changing in perceptual intensity across at least one of the blocks, ranging up to 50% of the magnitude estimation scale. Subject 1 reported the lowest intensity contour as changing across blocks but reported no difference in perceived magnitude for the higher intensities. Subject 2 reported a change in magnitude for all intensities and contacts (figure 9(a)).

#### 3.1.4. Different levels of muscle activation show no significant difference in R^2^ values for full and endpoint fits

For each fit method used with the muscle activation data, the *R*^2^ values across the 10 muscle-contact pairs were binned by the percent level of activation (10%, 30%, 50%, 70%, 90%) of the given muscle. No statistically significant differences were seen for the full fit or the endpoint fits. The full fit *R*^2^ values for the 5 levels of activation ranged from 0.994 to 0.997 while the endpoint fit *R*^2^ values ranged from 0.988 to 0.992. However, the any-two-point fit *R*^2^ values showed many significant differences, with the 90% condition being different from all other conditions ($p &lt; 0.01$). *R*^2^ values toward the middle of the activation range tended to be higher than the *R*^2^ values closer to 10% and 90%. Despite this trend, the median *R*^2^ values still had a narrow range of 0.983–0.989, which is unlikely to be functionally relevant.

### 3.2. Modeling

We treated each activation level as a separate equal intensity contour and fit the corresponding recruitment surfaces to the Weiss equation to calculate an *R*^2^ for each curve. Pooling these *R*^2^ values for the 99 motor SD curves with more than two (PW, PA) points across activation intensities in the motor model produced a median value of 0.9995 (IQR 0.9981, 1); the sensory model yielded a similar median of 0.9981 (IQR 0.9927, 0.9993) from 91 simulated SD curves.

**Figure 10.**
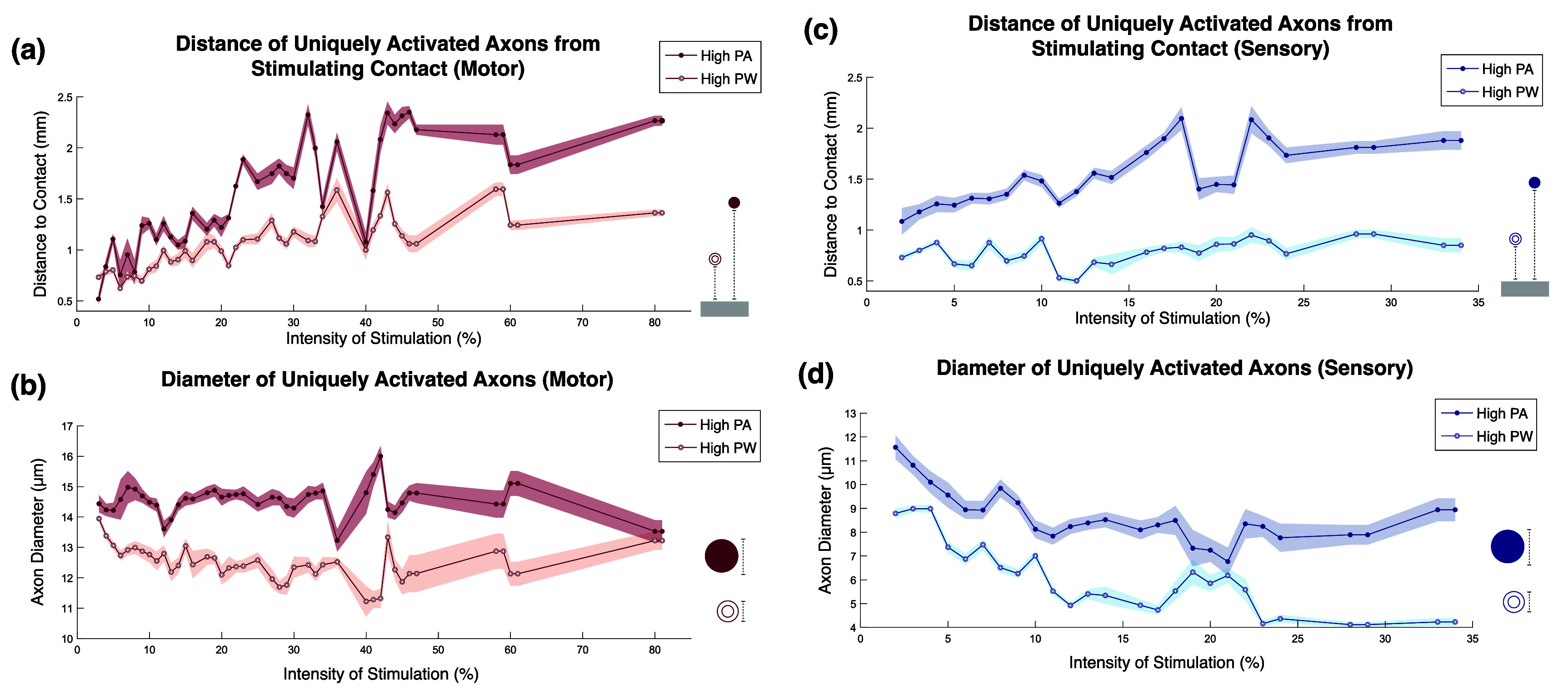
Axons uniquely activated by intensity-matched high-PA and high-PW stimulation. Axons that are uniquely activated by either high-PA (solid marker) or high-PW (open marker) stimulation represent the expected differences in activation on the vertical and horizontal ends of an SD curve. (a) and (c) High-PW stimulation consistently activates axons closer to the cathodic contact compared to high PA stimulation; (b) and (d) high-PW stimulation uniquely activates smaller-diameter fibers comparatively. Shaded regions represent 95% confidence intervals, with n ⩾ 30 for each point. Motor models are in red, and sensory models are in blue.

Across iso-intensities, two-sample t-tests show that the axon populations uniquely activated by high-PA and high-PW stimulation differ significantly in both diameter and distance to the stimulating contact. High-PW stimulation, or stimulation in the horizontal region of the SD curve, consistently activates axons closer to the simulated stimulating contact in both motor and sensory models (figures 10(a) and (c)), with 91.3% of sampled intensity pairs showing a significant difference in uniquely activated axon distance ($88.4\% \,p &lt; 0.01,\,2.9\% \,p &lt; 0.05$). Of the significantly different pairs, only one showed vertical region stimulation as activating axons closer to the contact. Similarly, high-PW stimulation, or stimulation in the horizontal region of the curve, activates smaller axons than high-PA stimulation across 95.7% of iso-intensity pairs (figures 10(b) and (d)) ($94.2\% \,p &lt; 0.01,\,1.4\% \,p &lt; 0.05$). Overall, these results confirm that the mean axon diameters and distances to the stimulating electrodes of the neurons uniquely activated by paradigms on opposing ends of the SD curve differ significantly between the two stimulation methods.

While there are systematic differences in the axons uniquely activated between high-PA and high-PW stimuli at the same iso-intensity, the two stimulation paradigms share many activated axons. We applied the Dice similarity coefficient (DSC) to characterize the similarity between the axons activated by the high-PA and high-PW iso-intensity stimuli. A DSC of 0 indicates no shared axons, while 1 indicates identical populations. DSC values generally rose with increasing intensity, and a Kruskal–Wallis test followed by Dunn-Šidák post-hoc comparisons revealed that the lowest-intensity data differed significantly from high-intensity data in both motor and sensory populations. For both motor and sensory axon models, the DSC below 5% intensity was about 0.75, while at higher intensities the DSC leveled off to 0.95.

## 4. Discussion

### 4.1. Accurate mapping of full functional ranges of muscle activation and perceived somatosensory intensity via SD curves

All clinically assessed intensity contours for both motor and perceived sensory neural activation fit the Weiss SD curve accurately (median full fit *R*^2^ > 0.98, minimum full fit *R*^2^ > 0.87). For muscle activation, these results provide clinical validation of the SD curve in a person with a spinal cord injury, adding to previously established literature in healthy populations [37, 39]. Further, the present study explores the accuracy of SD curves to describe muscle activation at levels above 60%, which is novel for all subject populations. The mapping of high-intensity muscle activation space is important for informing and improving the creation of sigmoidal recruitment curves and surfaces, which have dynamic curvatures at high intensities. The absence of a statistical difference between accuracy of fits across intensities for both full fits and endpoint fits validates the use of the SD curve to accelerate the process of generating full recruitment surfaces.

For perceived somatosensory activation, the application of an SD curve to suprathreshold percept intensities is novel. While SD curves have been shown to hold for suprathreshold CSAPs in healthy populations [37, 39], the human-in-the-loop aspect of determining multiple levels of perceptually equivalent neural activation adds a novel layer to generating the curves. Further, because Subject 2 does not have any muscles innervated by the median nerve in his residual limb, curves were able to be acquired at higher percept intensities without interference from muscle activation than might be possible in other participant populations. The expansion of perceptual SD curves beyond threshold also enables their use for exploring the PA-PW space for functional sensory restoration beyond baseline calibration and nerve health assessment [24, 26, 40]. Because the perceived magnitude of most curves varied across a session (figure 9(b)), we did not describe or compare the acquired SD curves as a percentage of the perceptual dynamic range. Regardless, the data showed a consistently high accuracy of SD curve fits at all tested perceptual intensities (figure 7(b)), validating their use to map the extrema of the perceptual intensity range.

### 4.2. Accurate estimation of SD curves in as few as two points

The clinical usefulness of the SD curve for describing the PA-PW space would be limited if many sample points were required to accurately define the curve. Because of the human response component of sensory percept characterization, each single-block nine-point sensory curve took about 10 min to acquire, and it took three hours to fully characterize four percept intensity curves. While motor data collection was more efficient than sensory due to the automated stimulation adjustments, the average of 3 min to describe a single curve and 16 min to map five curves for a single contact pair is still too inefficient for functional parameter space characterization.

We found no statistical difference between the goodness of fit of a sensory percept SD curve fit with 27 sensory data points and one solved with two endpoints, in either single-trial or trial-averaged methods. For muscle activation, there were statistical differences, but the median *R*^2^ difference of only 0.005 is unlikely to be functionally relevant. These results validate the use of two points to efficiently generate SD curves. This builds on Mogyoros’ clinical two-point SD curve model by showing that the accuracy of two-point solutions holds across all activation magnitudes and for perceptual intensity as well as compound action potentials [37]. Crucially, this approach identifies all (PW, PA) points that produce a given intensity, providing richer information in far less time than point-to-point sampling methods that rely on extrapolation. Reducing curve definition to two points makes mapping the full PA-PW space clinically and experimentally feasible.

### 4.3. Dependency of two-point SD curve accuracy on point sample position

In alignment with Alavi’s simulation-based findings [42], two-point fits were statistically significantly superior when sampled at the extrema of the parameter space in both motor and sensory activation (figure 7). These results show that point position influences accuracy in clinical SD curve fits, and that there is a need for a method to assess if samples are close enough to the curve extrema to produce a quality SD curve.

To generalize point position independent of stimulator range or tissue impedance, the any-point curve fit distribution was expressed in terms of the ratio of the SD curve derivative at the points selected (figure 8, equation (2)). As expected, point pairs with more disparate derivatives, i.e. positioned on opposite sides of the elbow of the SD curve, produce better fits than pairs with similar derivatives. While the *R*^2^ fall-off is a continuum, cut-off values were based on the 25th percentile and lower bound of the endpoint *R*^2^ distributions for each respective modality. The any-point fits differ from the endpoint distribution above SRs of 0.68 and 0.34 for motor and perceptual sensory curves, respectively. For the sensory distribution (figure 8(b)), there are median *R*^2^ values within the endpoint distribution up to a SR of 0.68, but they are interspersed with lower *R*^2^ solutions, indicating a higher risk of a poor fit if the points are sampled in that space. Overall, a pair of points are likely to produce an accurate SD curve if the ratio of their slopes is less than 0.34 for perceptual sensory applications or 0.68 for motor applications.

With respect to whether trial-averaging the point pair is needed to generate an accurate SD curve, Forst *et al* found that SD curve fit error tended to decrease with three to four trials per point in sensory surface stimulation [24]. However, these results did not consider the position of the sampled points. For the perceptual sensory SD curve fits, there was a significant difference between the trial-averaged and single-trial distributions when any two points were sampled but not when endpoints were sampled (figure 7(b)). Further, when expressed in terms of SR, the trial-averaged any-point distribution matched the single-trial distribution at low SRs, with the same *R*^2^ fall-off at 0.34. However, trial-averaged samples are more robust at higher SRs; while the last *R*^2^ value within the endpoint distribution occurs at a SR of 0.68 for single-trial point pairs, there are trial-averaged point pairs with *R*^2^ values within the endpoint distribution up to 0.81. Overall, if the sampled point pair have a SR of less than 0.34, trial-averaging is not a necessary step to ensure a quality sensory SD curve fit. Otherwise, trial-averaging may have a positive impact on fit quality when sample points are closer together.

#### 4.3.1. Summary: recommendation for how to efficiently collect SD curves

To most efficiently and accurately acquire an SD curve, we recommend sampling two iso-intensity points, one each at the highest possible PA and the highest possible PW allowed by the interface and stimulator. With respect to sensory sampling, ask participants to compare the intensity of the two points rather than relying on magnitude estimation to maximize the precision of the curve.

After collecting the two equal intensity points, the point pair should be used to solve the Weiss equation (equation (1)). To ensure the collected points are far enough apart on the curve for an accurate estimate, calculate the derivative of the resulting SD curve at the location of the two sample points (equation (2)). Calculate the SR between the two points (equation (3)), and check that the SR is less than 0.34 for sensory curve formation and 0.68 for motor curve formation. If the SR is greater than these cut-offs, consider (a) moving the solver points further toward the extremes of the stimulation space, if possible; (b) taking multiple trials at the already established point positions and trial-averaging the pulse parameters before re-solving for the Weiss equation; or (c) sampling more (PW, PA) points in between the solver points and performing a non-linear fit.

### 4.4. Using the SD curve to define functional stimulation parameter regions

In both sensory and motor PNS applications, SD curves can efficiently outline the region of stimulation that provides functional benefit. These regions are bounded by several factors, including comfort, the differing thresholds at which muscle contraction, sensation, and pain are first activated, and the maximum activation level each can reach. Each of these functional limits can be defined by a single SD curve. Obtaining an SD curve for each limit therefore defines the complete boundary of the functional region of a neuroprosthesis.

For FES neuroprostheses, it is important to avoid painful or distracting sensory fiber activation while evoking the broadest range of forces to perform functional tasks. For sensory neuroprostheses, unintended muscle activation can affect EMG control of a prosthetic limb or elicit conflicting proprioceptive or skin stretch percepts. Unwanted muscle activation also limits the upper portion of the functional sensory dynamic range, as users experience uncomfortable muscle cramping at a lower current than their maximum comfortable sensory percept.

Stimulation regions that evoke muscle activation, non-noxious somatosensory percepts, and pain can be detangled by acquiring response-specific SD curves. Using this method, the complete region of stimulation parameters that avoid unwanted axon activation can be quickly and easily defined. This approach enables rapid identification of safe, functional regions for stimulation without exhaustive point-by-point sampling, making it practical for clinical calibration of many different types of interfaces.

### 4.5. Using the SD curve to three-dimensionally characterize activation intensity as a function of both PW and PA

#### 4.5.1. Recruitment surface construction method

Because of the SD curve’s accuracy and robustness, it has potential for fully describing all axonal activation that occurs within a given functional region. We propose a novel method to construct recruitment surfaces, which are complete 3D models of the axonal activation that occurs within the 2D region defined by modulating both PA and PW stimulation parameters. Recruitment curves, and to a lesser extent recruitment surfaces, are standard in motor FES where objective measures of intensity such as EMG or force are plotted as a function of modulating either or both PA and PW. They are less common in sensory applications where perceived intensity is subjective and data collection is slower. However, psychophysical scaling can express two intensities relative to each other, enabling intensity to be used as a *z*-axis.

For each recruitment surface, the proposed method acquires a PW-modulated recruitment curve at the maximum PA value and PA-modulated curve at the maximum PW value. SD curves would then be solved using equal-intensity points on the two recruitment curves across the full range of axonal activation. If multiple muscles, percept locations, or percept qualities are being tested, one recruitment curve would be simultaneously collected for each output.

This novel characterization method would require an order of magnitude fewer points than a full procedural sampling of the space and therefore make it practical to use the entire stimulation space rather than the few slices PW or PA modulated curves have historically provided.

#### 4.5.2. Benefits of recruitment surface characterization

Knowledge of the full surface facilitates improved neural output resolution and ensures that the full dynamic range is accessible. By modulating both PA and PW with respect to the gradient of the surface, the resolution of intensity changes can be more precisely controlled. A stimulation adjustment nearly parallel to an SD curve will yield subtler changes in output intensity compared to an equivalent adjustment oriented perpendicular to the same SD curve. In motor applications, recruitment curves with shallower slopes have long been sought after to provide better resolution of muscle activation [4–7]. In sensory applications, by moving across a multi-dimensional space, finer gradations in percept intensities are accessible that were previously within a stimulator step size, supporting applications such as object recognition and compliance detection [8]. However, in both modalities, overly shallow slopes prevent access to the full dynamic range. Currently, multiple recruitment curves are often needed to find parameters that evoke the full range of activation with a sufficiently shallow slope. By characterizing the full stimulation space, it is possible to automatically find the shallowest path from threshold to maximum, even if that path requires PW and PA to be modulated independently or nonlinearly.

Efficient characterization of recruitment surfaces for multiple neural response types also facilitates research into joint motor and sensory topics such as proprioception. For example, the effect of PW on the strength of the H-reflex with respect to the M-wave is an active area of research, and utilizing both motor and sensory SD curves can aid in the exploration of H-reflex activation while maintaining a constant M-wave [12, 60, 61].

For sensory neuroprostheses, the shape of a recruitment surface also provides insight into multi-dimensional just noticeable difference (JND) measurements. Previous studies comparing the relative impact of different stimulation parameters on perceived intensity have evaluated the Weber fractions of each parameter at one or two points [27, 40]. However, the relative effect of PA and PW on intensity varies along the SD curve. Two-dimensional JNDs that examine the perceptual intensity gradient across a recruitment surface would more accurately evaluate parameter impact.

### 4.6. Differential axon population activation in different regions of the PA-PW space

#### 4.6.1. *In silico* basis of differences in axon populations

The modeling results indicate that high-PA and high-PW stimuli on the same SD curve activate many of the same axons, but that the subsets of axons that are unique to each end of the curve systematically differ. In the more horizontal regions of the curve (high-PW), stimulation consistently activates axons closer to the electrode and smaller in diameter. Conversely, in the more vertical regions of the curve (high-PA), the uniquely activated axons are further away from the contact and include more large-diameter fibers (figure 10). These results are consistent with previous modeling and experimental data that state that the difference in threshold of axon activation is the smallest between diameters at a high-PW compared to low-PW stimulation [9, 11]. This effect is likely due to currents between nodes of Ranvier within an axon, which are greater in larger diameter axons [11]. Whereas these previous studies have demonstrated the differences in axon activation in single axons across PWs, our results extend these findings to a full population of axons within a human-derived nerve model. Notably, the same systematic differences in activated axon populations appear in both motor and sensory models.

A DSC analysis with Kruskal–Wallis tests confirms that the lowest-intensity SD curves activate more distinct axon populations than those at higher intensities in both sensory and motor models. This suggests that alternating between stimuli along an SD curve at low intensities may produce more distinguishable sensations in sensory stimulation and recruit different motor units compared to high-intensity stimuli. Clinical validation is needed to determine how unique the two axon populations need to be to constitute a meaningful difference in activation.

#### 4.6.2. Motor applications of differences in axon populations

If the differences in motor unit activation on opposing sides of the SD curve prove to be functionally significant, full characterization of the parameter space should enable more dexterous movements and decreased fatigue. For surface and implanted motor neuron stimulation, multiple muscles are often activated by a single electrode contact, and the relationships between these muscles can covary in unpredictable ways. Single contact muscle selectivity could be maximized by utilizing the differences in muscle activation in the vertical and horizontal regions of the SD curve. Based on our results, stimulating in the horizontal region may recruit axon populations that are closer together, leading to more anatomically-aligned muscle activation [62]. This increased ability to more independently modulate individual muscles should allow for more accurate and dexterous movements.

Following Henneman’s size principle, smaller alpha motor neurons innervate slow-twitch muscle fibers while larger efferents innervate fast-twitch muscle fibers [63]. Thus, stimulation in the horizontal region of the curve may also more closely mimic natural recruitment by activating smaller fibers earlier, yielding smoother, more fatigue-resistant contractions for FES. Additionally, interleaved stimulation of non-identical motor unit populations that perform the same movement have been shown to decrease fatigue while maintaining activation strength in intramuscular [13], intrafascicular [14], and cuff electrodes [15]. Modulating PA and PW independently would make it easier to activate motor unit populations with less overlap, therefore increasing stamina.

#### 4.6.3. Sensory applications of differences in axon populations

Applying the modeling results to sensory neuroprostheses, differential firing based on axon diameter may help select between large proprioceptive fibers, efferents, and somatosensory afferents [64]. Further, axon distance from the stimulating contact is clinically relevant because fibers are generally arranged somatotopically such that those innervating the same muscle or neighboring areas of skin are grouped together [65, 66]. Given that the activation of a single afferent fiber is perceivable [67–69], we would expect differences in the fiber populations recruited at each end of an SD curve to be perceivable in sensation location or quality. The manipulation of these percept features independent of intensity would be highly impactful, as intensity covaries with these characteristics in standard PNS paradigms [8, 28].

Regarding percept location, results were mixed in this study as to whether the reference stimulus in the horizontal region of the curve and trial stimuli in the vertical region elicited different sensations. Subject 1 reported sensation on the thumb and index finger throughout the testing. However, for the curve just beneath muscle contraction, which the participant rated as mid-intensity in magnitude, the reference stimulus in the horizontal region elicited a sensation on the intermediate phalanx of his third finger that diminished and eventually disappeared in the trial stimuli as PA increased into the vertical region. The participant also noted that for the percept on the middle finger to appear during the higher PA trial stimuli, he had to increase PW such that the percept on the thumb was significantly stronger than that elicited by the reference stimulus. Ultimately, Subject 1 decided to match the intensities on the thumb and index fingers between reference and trial stimuli to construct the curve. Subject 2, however, did not report any differences in percept location within a curve. Further study with a larger participant pool is needed to fully assess the potential for SD curves to modulate location while holding intensity constant.

### 4.7. SD curve robustness to perceptual sensory adaptation

The absence of a significant difference in any sensory SD curve across three hours of data collection indicates the robustness of SD curves to sensory perceptual adaptation (figure 9(b)). All contacts showed evidence of perceptual adaptation, with an average increase in perceptual threshold of 0.075 mA at 250 *µ*s, in addition to changes in estimated magnitude (figure 9(a)). Applicably, because the quality and values of the curve fits are shown to be unaffected by perceptual drift, SD curves do not need to be recalculated across hours of use. Instead, to account for adaptation, a single point from each existing curve should be sampled to update the curve’s perceptual magnitude. New curves should only need to be generated for high intensities that were previously above the threshold for discomfort. The resilience of the SD curve solution to perceptual drift increases its utility in both take-home closed-loop neuroprostheses as well as in-lab experiments, as reassigning the curves a new perceptual magnitude takes only seconds as opposed to minutes of resampling.

Mechanistically, the maintenance of each SD curve across the two-dimensional space may point to perceptual adaptation as a centrally mediated process rather than a change in peripheral nerve dynamics. This aligns with previous hypotheses on the nature of tactile temporal inhibition [70, 71]. As demonstrated in the *in silico* data, intensity-matched stimuli at high-PA and high-PW tend to activate neural populations that contain a subset of unique fibers. Although we have not proven these trends clinically beyond Subject 1’s anecdotal percept location changes, if adaptation were peripherally mediated, we would expect the reference stimulus on the horizontal region of the curve to adapt faster than trial stimuli in the vertical region due to repeated activation. However, the absence of any shift in the generated curve demonstrates that adaptation is occurring equally to all subsets of that contact’s neural population. This points to adaptation occurring proximal not only to mechanoreceptors as previously shown [71] but to peripheral nerves as a whole. To better support this theory, further research is needed to prove that different neural populations are activated at different parts of the SD curve in a clinical model.

### 4.8. Limitations

#### 4.8.1. Sample size

While the motor and sensory analyzes in this study consist of 2312 and 486 sampled points, respectively, the number of participants that the data were acquired from was limited. In the motor analysis, data were only acquired from a single participant with 5 nerve cuff electrodes (2 contacts on each) tested within that participant’s right arm and shoulder. Despite this limited sample size, the fact that the our low intensity and all-points-fit motor data aligns with previously published modeling and physiological data provides some confidence that the motor findings are likely to be broadly applicable [36, 37, 39]. While patient specific factors such as age, sex, and conditions like ischemia have been shown to affect the parameters of the SD curve [72, 73], this study focuses on the predictability of the shape. Changes in the shape of the SD curve itself has only been observed when stimulating partially denervated muscles directly rather than through the nerve stimulation that was done in this study [74]. In the sensory analysis, data were acquired from two participants with different levels of experience with sensory stimulation studies. One nerve cuff electrode was tested each in Subject 1 (1 contact) and Subject 2 (3 contacts). This limited subject population is due to the rarity of the relevant injuries and the intensive nature of the implantation and research. While these results should be validated in more people, we posit that the findings will be generalizable given they align with the larger body of SD curve literature and are consistent across all 50 motor curves and 16 sensory curves.

For the qualitative data collected from sensory participants such as magnitude estimation and percept location, the small sample size limits the strength of some of the proposed applications due to the differential experience between Subject 1 and Subject 2. Subject 1 has eight years of experience with PNS studies and has accordingly developed a comparatively precise system for how he reports percepts. For example, Subject 1 commonly relates his estimated magnitudes to increases in percept areas, so he quickly noticed when different ends of the curve produced slightly different percept areas at equal intensities. Data collection for Subject 2 took place during his first year enrolled in the study, and as a result his system for reporting percepts was less consistent. Future studies with more participants are needed to further explore the application of SD curves to sensory percept manipulation.

#### 4.8.2. Differences in target reference consistency between motor and sensory experiments

Even though no significant temporal shifts were detected in the sensory contour data, 60% of the equal muscle activation contours showed a significant difference ($p &lt; 0.05$) between data collected in different experimental blocks. This likely results from differences in the target references between the two modalities. For the motor experiments, each target EMG value was determined at the beginning of the session as a percentage of the maximum EMG recorded for that muscle. This means that any changes in neural response or muscle activation that occurred over the course of data acquisition were not reflected in this target value. For the sensory experiments, the target reference was the live perceived intensity at a specific $\left( {{\text{PW, PA}}} \right)$ point, meaning any changes in sensory perception were reflected in both the reference and sampled points. It is most likely that, in the same way that the sensory curves stayed consistent while the reported magnitude changed across blocks (figure 9), the differences seen between blocks in the motor results could have been controlled for by renormalizing target EMG to an updated maximum EMG throughout the session.

## 5. Conclusion

This work provides a clinical and *in silico* foundation for the application of SD curves to characterize and improve outcomes of PNS for the control of muscle activation and restoration of somatosensation. The clinical data demonstrates that the SD curve is an accurate model of muscle activation and sensory intensity contours across the full functional range of activation. Our analysis of different fitting techniques shows that solving the SD curve equation with the two points that are furthest into the horizontal and vertical regions of the curve provides significantly better curve fits than arbitrary combinations of two points. Further analysis shows that two-point SD curve solutions with SRs under 0.68 for motor and 0.34 for sensory are within the expected distributions of the endpoint solutions. The *in silico* modeling shows that one can expect to see greater activation of axons that are further away from the electrode and axons that are larger in diameter when stimulating in the vertical region of the SD curve as compared to the horizontal region.

The combination of these findings suggests many benefits of using the SD curve for the characterization of PNS and potentially other stimulation methodologies. For motor FES, characterizing the full stimulation space with SD curves could help create neuroprostheses that have better muscle selectivity, fatigue resistance, and fine motor control. For sensory PNS, SD curves could facilitate unique percept generation, agile adaptation adjustment, and high-resolution intensity modulation. For both modalities, the SD curve can be used to quickly define a comfortable stimulation region and avoid unwanted activation of the other modality. We also believe that this framework could have useful applications in other types of neurostimulation such as surface stimulation and spinal cord stimulation. Overall, the efficiency of this framework increases the clinical feasibility of multidimensional parameter characterization for advanced, patient-specific neuromodulation outcomes.

## Data Availability

The data cannot be made publicly available upon publication because they are not available in a format that is sufficiently accessible or reusable by other researchers. The data that support the findings of this study are available upon reasonable request from the authors.
